# Informative presence and observation in routine health data: A review of methodology for clinical risk prediction

**DOI:** 10.1093/jamia/ocaa242

**Published:** 2020-11-09

**Authors:** Rose Sisk, Lijing Lin, Matthew Sperrin, Jessica K Barrett, Brian Tom, Karla Diaz-Ordaz, Niels Peek, Glen P Martin

**Affiliations:** 1 Division of Informatics, Imaging and Data Sciences, School of Health Sciences, University of Manchester, Manchester, United Kingdom; 2 MRC Biostatistics Unit, University of Cambridge, Cambridge, United Kingdom; 3 Cardiovascular Epidemiology Unit, Department of Public Health and Primary Care, University of Cambridge, Cambridge, United Kingdom; 4 Department of Medical Statistics, London School of Hygiene and Tropical Medicine, London, United Kingdom; 5 NIHR Biomedical Research Centre, Manchester Academic Health Science Centre, University of Manchester, Manchester, United Kingdom; 6 Alan Turing Institute, University of Manchester, London, United Kingdom

**Keywords:** clinical prediction model, electronic health records, informative observation, informative presence

## Abstract

**Objective:**

Informative presence (IP) is the phenomenon whereby the presence or absence of patient data is potentially informative with respect to their health condition, with informative observation (IO) being the longitudinal equivalent. These phenomena predominantly exist within routinely collected healthcare data, in which data collection is driven by the clinical requirements of patients and clinicians. The extent to which IP and IO are considered when using such data to develop clinical prediction models (CPMs) is unknown, as is the existing methodology aiming at handling these issues. This review aims to synthesize such existing methodology, thereby helping identify an agenda for future methodological work.

**Materials and Methods:**

A systematic literature search was conducted by 2 independent reviewers using prespecified keywords.

**Results:**

Thirty-six articles were included. We categorized the methods presented within as derived predictors (including some representation of the measurement process as a predictor in the model), modeling under IP, and latent structures. Including missing indicators or summary measures as predictors is the most commonly presented approach amongst the included studies (24 of 36 articles).

**Discussion:**

This is the first review to collate the literature in this area under a prediction framework. A considerable body relevant of literature exists, and we present ways in which the described methods could be developed further. Guidance is required for specifying the conditions under which each method should be used to enable applied prediction modelers to use these methods.

**Conclusions:**

A growing recognition of IP and IO exists within the literature, and methodology is increasingly becoming available to leverage these phenomena for prediction purposes. IP and IO should be approached differently in a prediction context than when the primary goal is explanation. The work included in this review has demonstrated theoretical and empirical benefits of incorporating IP and IO, and therefore we recommend that applied health researchers consider incorporating these methods in their work.

## INTRODUCTION

### Background and significance

Clinical prediction models (CPMs) estimate the risk that a patient currently has (diagnostic), or will develop (prognostic), an outcome of interest based on known clinical and patient measures. Such risk models can guide clinical decision making, among other uses.

**Figure 1.**
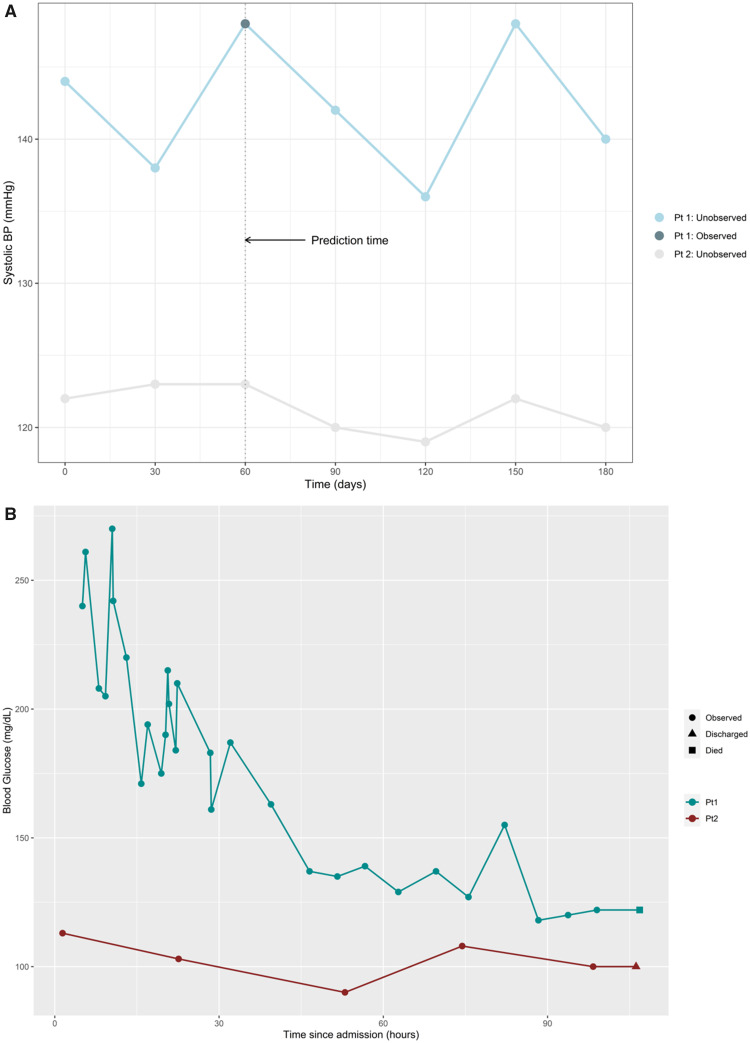
(A) An illustration of informative presence and how this could impact the information available at prediction time. We see the longitudinal pattern of blood pressure for 2 patients, with both their observed and unobserved values shown. Patient 1 has 1 single observed value of systolic blood pressure (BP), and this happens when their BP was at its highest. Patient 2 has no observed values, but their BP remains in the normal range—either the patient or clinician saw no clinical need to take a blood pressure measurement at any time. (B) An illustration of informative observation, taken from the MIMIC (Medical Information Mart for Intensive Care) dataset.^4^ Patient 1 has many more in-hospital measurements of blood glucose than patient 2 throughout their intensive care unit admission, likely due to the fact that their blood glucose is much higher and much more variable than patient 2. A more severe condition often means more intense monitoring.

Widespread adoption of electronic health records (EHRs) facilitates the development of CPMs,^1^ as detailed clinical and patient information is collected through routine healthcare contacts. Such rich longitudinal information provides long-term patient follow-up without the need to recruit patients and conduct regular follow-up visits. The analysis of routinely collected data is not, however, without challenge. Observation times are not prespecified as they would be in a typical research study (eg, in a prospective cohort study with scheduled follow-up visits). Instead, data are collected opportunistically, in which patient and clinician decisions directly dictate whether we observe clinical biomarkers and patient information.^2^ For example, general practitioner visits occur more frequently during periods of ill health,^3^ and only information relevant to the particular consultation will be recorded. Equally, during inpatient care, clinicians will adapt their monitoring frequency to the changing needs and condition of the individual patient (see Figure 1).

We refer to the process by which visits, and hence measurements, occur as the observation process (also known elsewhere as the visiting or monitoring process)*.* We define 2 key properties that an observation process may have, when presence of data is informative:

1. Informative presence (IP) (Figure 1A): The presence or absence of a patient’s data at any given time point carries information about their health status.
2. Informative observation (IO): The timing, frequency, or intensity (rate) of a patient’s longitudinal pattern of observation carries information about their evolving health state. See Figure 1B for an example.

IP is challenging from a statistical perspective as it implies a missing not at random process. IP is, however, conceptually different from missingness, as in the former, there was never any intention of collecting the data at a particular visit. IP has previously been defined elsewhere,^5^^,^^6^ with Phelan et al^5^ discussing how interactions contained within EHRs are informative with respect to patient health.

IO is the continuous time generalization of IP: a longitudinal visiting (at time t) not at random process, defined as “given data recorded up to time t, visiting at time t is not independent of outcome at time t.”^7^ By generalizing the definition of IP above, one can draw value from how frequently a patient is observed over time. This is especially true when no schedule exists dictating when or how often visits should occur; we therefore focus on what an individual’s longitudinal observation process could tell us about their condition.

A recent review of CPMs developed using routinely collected data revealed an apparent lack of understanding of, or proper handling of, IP and IO.^1^ Moreover, much of the existing methodological literature in this area has focused on IP and IO only in the context of effect estimation (ie, in causal or associational studies),^8–14^ and has generally viewed it as a “nuisance” (ie, a phenomenon that potentially biases effect estimators and therefore needs to be corrected for in the analysis). However, when developing a CPM, the primary focus is on achieving good predictive performance; predictor effect estimation is less important.

Instead, one could view IP and IO as opportunities to draw information from the EHR that is not explicitly recorded. In this article, we focus on informative measurement patterns in the predictors, and we do not discuss presence or absence of outcome data. Agniel et al^15^ demonstrated how the timing of a lab test better predicts mortality than the actual result of the test. Others have illustrated how incorporating the presence or absence of a particular test for an individual into a CPM can improve its accuracy.^16–18^

### Objectives

This article aims to review the literature on methodology allowing CPMs to utilize IP or IO, both in overcoming some of the aforementioned challenges, and in harnessing information within informative measurement patterns. In doing so, we also highlight outstanding areas of methodological work that should be prioritized. Finally, we summarize existing software packages capable of implementing the methodology.

## MATERIALS AND METHODS

The strategy employed in this review loosely follows a scoping review framework.^19^ Our protocol has been registered on the Open Science Framework.^20^

### Search strategy

We searched MEDLINE, Embase, and Web of Science for relevant articles using prespecified search terms. Further details of the full search strategy (including search terms and an additional snowballing stage) can be found in the Supplementary Appendix and the published protocol.^20^

### Study selection

We had the following inclusion criteria: any article presenting a method that allows CPMs to incorporate IP or IO. We excluded articles that applied existing methods that had already been published elsewhere, and included those earlier publications instead, nonmedical areas of application, IP and IO in outcome measures, and methods that handle sample selection bias, imputation or censoring only. See the Supplementary Appendix for further justification of these exclusions.

We do not include textbooks within the review; while this could mean we miss some relevant literature, searching within textbooks is not widely feasible. Additionally, we believe that most methodological development in this area will be published in original research articles rather than textbooks.

Two independent reviewers (R.S., L.L.) conducted a 2-stage screening process. Titles and abstracts were screened first, and full texts of remaining articles were reviewed at the second stage. Reviewers met regularly to track agreement. Systematic differences were translated into new inclusion and exclusion criteria, in consultation with a third reviewer (G.P.M.).

Primarily, we extracted information regarding the modeling method employed and any reported advantages and disadvantages. We also extracted information on the form of the observation processes, predictors, and outcome, including any clinical use cases presented.

## RESULTS

**Figure 2.**
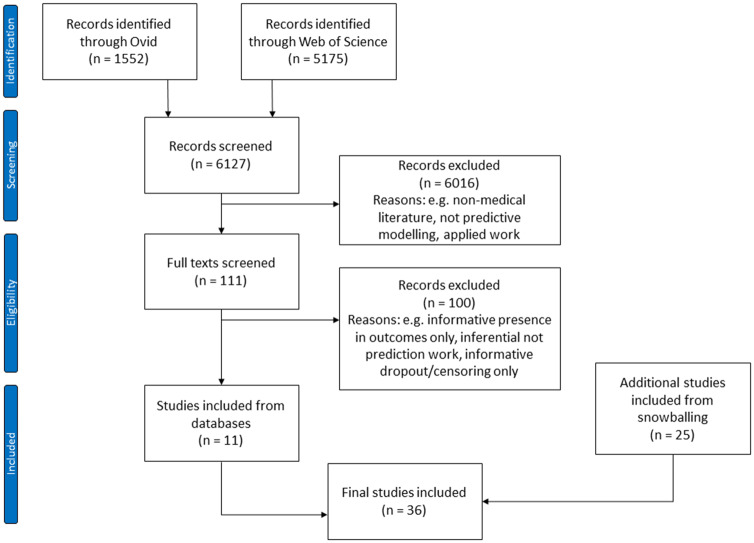
PRISMA (Preferred Reporting Items for Systematic Reviews and Meta-Analyses) flow diagram showing the various screening stages and reasons for exclusion at each stage.

Our database searches identified 6127 studies, of which 111 were retained for full text screening. Eleven of these were deemed eligible for inclusion. We identified a further 25 articles through forward and backward citation searching, giving a final set of 36 included articles (Figure 2).

Throughout this section, we will illustrate each method with the following notation. Consider a binary outcome $Y\left(t\right)$ (or Y if only observed once) for patients i=1,…,n, at time t, where Y=1 denotes that the event occurred, with marginal probability $P\left[Y=1\right]$. Define a potentially time-varying continuous covariate process X(t), with potential realizations $x_{ij}$ for i=1,…, n and $j=1,\ldots m_{i}$, or simply $x_{i}$ if X is not time-varying. The timing of the j^th^ realization of $X\left(t\right)$ is $t_{ij}\in \;R^{+}$. Denote R=1 if $X\left(t\right)$ is ever observed, and R=0 if not. Define $r_{ij}$ = 1 if the covariate process is observed at time $t_{ij}$. We assume that Z is a completely observed time-invariant covariate. g(.) represents a link function (eg, the logit function).

Broadly, the methods in this article cover the 3 scenarios described in Table 1. To illustrate the prediction scenarios and methods, we consider a simplified version of the Sequential Organ Failure Assessment score,^21^ used to predict mortality in critical care, assuming that the only predictors in the model are bilirubin and blood pressure. Of these 2 predictors, we assume that blood pressure is completely observed for all patients, and that bilirubin is informatively observed, as it has been shown to be within critical care. Depending on the specific scenario, it may be a one-time point observation, or a longitudinal process.^17^

**Table 1.** A description of different prediction scenarios, covering cross-sectional vs longitudinal predictors and outcomes

Scenario	Scenario name	Description	Example (SOFA)
S1	Cross-sectional prediction	Interest lies in obtaining a single prognostic estimate (prediction) using a single value for each predictor.	Use values of bilirubin and BP obtained upon ICU admission to predict in-hospital survival (binary).
S2	Cross-sectional prediction with longitudinal predictor measurements	Interest lies in obtaining a single prognostic estimate but using the longitudinal history of predictor values.	Use all repeated lab tests obtained throughout inpatient admission for bilirubin and BP to predict in-hospital survival.
S3	Longitudinal prediction with longitudinal predictors and outcomes	Interested in prognostic estimates at multiple time points, potentially using the longitudinal history of predictor values.	Use all repeated measures of BP and bilirubin obtained throughout inpatient and ICU admission to predict survival at multiple future time points.

BP: blood pressure; ICU: intensive care unit; S: scenario; SOFA: Sequential Organ Failure Assessment;

There exists a breadth of methodological literature covering scenario 2 (S2) (without accounting for IP and IO), which has recently been synthesized by Bull et al.^22^ We therefore focus on modeling strategies that have specifically been proposed or extended to accommodate IP or IO.

### Identified approaches to handle IP and IO

We identified 3 broad categories of method based on the included articles: (1) methods that incorporate IP or IO through derived predictors; (2) methods for modeling under IP; and (3) methods that incorporate IP or IO using latent structures. Within these 3 categories, we identified 8 modeling strategies. A summary of the methods can be found in Table 2. Table 3 summarizes the advantages, disadvantages, software, and assumptions for each method—here, the reported advantages and disadvantages were inferred by the research team because they are not consistently mentioned in the included literature. A summary table at article level can be found in Supplementary Appendix 3.

**Table 2.** Descriptive summary table of methods, detailing when each method may be appropriate and how it would work with the running example of a simplified SOFA score

Modeling approach	Broad category	Refs	Scenario(s)	IP or IO	Description	Example
Missing indicators & Separate class	Derived predictors	^16^ ^,^ ^23–30^	S1	IP	Creating a binary indicator, representing presence/absence of a predictor at a given time point or in a given window	Create a binary indicator taking 0 when bilirubin is observed, and 1 if missing. Enter this as an additional predictor alongside observed bilirubin and BP.
Summary measures	Derived predictors	^15^ ^,^ ^24^ ^,^ ^31–44^	S2	IO	Summarizing the observation process into a single variable, eg, counting visits, rates of visits over a window, weighted counts	Count the number of times bilirubin has been measured over the first 24 hours of each ICU admission. Enter this count as an additional predictor in the model.
Pattern-specific models	Modeling under informed presence	^45^ ^,^ ^46^	S1	IP	Derive separate models for each missingness pattern	Develop models for: bilirubin and BP observed, and only BP observed
Likelihood-based methods	Modeling under informed presence	^47^ ^,^ ^48^	S1	IP	Incorporating missingness mechanism into maximum-likelihood estimation of parameter estimates	Bilirubin is missing not at random. Estimate model parameters using method-of-weights and EM algorithm.
Similarity measures	Derived predictors	^49^	S2	IO	Calculate similarity between target patient and all others, based on predictor values and measurement timings. Develop models separately for “similar” groups of patients.	Develop separate models amongst cohorts of patients with similar bilirubin, BP and timings of those measures.
Latent variable	Latent structures	^50^ ^,^ ^51^	S1, S3	IP	Outcome can be partially latent, and the observation process infers the latent state.	The occurrence of a bilirubin measurement is used to infer patient state in a hierarchical model.
HMMs	Latent structures	^52^ ^,^ ^53^	S3	IO	Outcome is a partially latent process, and the observation process infers the state at any time.	The intensity of bilirubin measurements over the course of a patient's admission infers their severity at any time point.
Joint modeling/shared random effects	Latent structures	^54–56^	S2, S3	IP and IO	Model the outcome, predictor and observation processes separately, but join them through random effects shared across the models.	Model the number of times bilirubin is measured throughout the admission as a point process, the repeated measures of bilirubin using a linear mixed model, and the binary outcome using a logistic regression. Link these via at least 1 shared random effect across the models.

BP: blood pressure; HMM: hidden Markov model; ICU: intensive care unit; IO: informative observation; IP: informative presence; S: scenario; SOFA: Sequential Organ Failure Assessment.

**Table 3.** Summary of (subjective assessments of) advantages, disadvantages, software, and assumptions for each method described in this review

Modeling approach	Advantages	Disadvantages	Software	Assumptions
Missing indicators & Separate class	Straightforward Flexible Low computational cost Easy to communicate	Potentially doubles no. of predictors Too simplistic for complex relationships between missingness and outcome Assumes discrete time intervals	Easily applied in common statistical software	Assumes absence is a proxy for some unmeasured patient feature Linear relationship with outcome
Summary measures	Straightforward Flexible Low computational cost Easy to communicate	Generalizability of models across centers may be questioned May fail to capture complex relationships between observation process and outcome	Easily applied in common statistical software	Assumes observation process is a proxy for some unmeasured patient feature Largely assumes linear relationship with outcome
Pattern-specific models	Straightforward Flexible	Number of models becomes large as no. of predictors increases	Easily applied in common statistical software	No assumptions placed on how missingness relates to observed or unobserved variables Assumes same functional form for all pattern-specific models
Likelihood-based methods	Also allows for imputation	Computationally intensive	None provided	Assumes absence is related to the unobserved value
Similarity measures	Flexible	Computationally intensive	None provided	None provided
Latent variable	Improved performance over methods not incorporating informative presence	Computationally intensive	R code provided by Coley and Hubbard	Association between outcome and observation process is captured through latent variable and other predictors
HMMs	Using a Hawkes process for intensity allows for time-varying intensity	Complex and computationally intensive	None provided	Assumes longitudinal predictors are normally distributed
Joint modeling/shared random effects	Flexible to different forms of outcome and observation process	Complex Computationally intensive Often requires independence assumption between processes given random effects	Frailtypack in R, WinBUGS, merlin in STATA for flexible user-defined models.	Assumes processes (outcome, observation) are independent conditional on random effects Existing methods assume constant intensity of observation

HMM: hidden Markov model.

### Category 1: Derived predictors

The methods described in this section address IP or IO by deriving some representation of the observation process and including this as a separate predictor in the model to exploit the informativeness for predictive value. These approaches tend to be straightforward and have been proposed to handle both IP and IO. However, attention must be paid to the intended use of the final model, particularly where the model will be applied in clinical settings different to the one in which it was developed. Where measurement protocols change across different settings, these models may lack generalizability when transported to a new setting.^57–59^ This should not be a concern where the development and application settings remain the same.

#### Missing indicators or separate class

The missing indicator approach^16^^,^^23–30^ handles IP in a straightforward manner, by deriving a binary variable that indicates whether a predictor has been observed at a specific time (IP) or over a defined window of time. The indicators enter the prediction model as a separate predictor alongside other patient and clinical information. For example, if a prediction model requires an entry for bilirubin but this test has not been conducted, then a missing indicator would be included as a predictor with value 1 (or 0 when observed). For categorical variables, a separate “missing” category could instead be created.

Because most prediction models require a value for every predictor, the missing indicator approach is usually combined with imputation at both model development and prediction time (not necessary for categorical predictors with a separate class).The missing indicator approach results in a model of the form:

(1) $$g\left(P[Y=1|X,\;Z]\right)=\beta_{0}+\;\beta_{1}X+\;\beta_{2}Z+\gamma R$$

for continuous predictors within cross-sectional prediction (S1).

Similarly, for a categorical predictor $x_{i}$ with k categories, then the missing indicator approach would set $x_{i}\in \;\{{\mathrm{Cat}}_{1},\;\ldots ,\;Cat_{k},\;\text{Missing}\}$ and our model would be

(2) $$g\left(P[Y=1|X,\;Z]\right)=\beta_{0}+\;\beta_{1}X+\;\beta_{2}Z$$

The previous 2 equations could be combined to consider prediction models with both continuous and categorical predictors. Alternatively, missing indicators and separate classes have been well developed in tree-based prediction algorithms.^28–30^

Including a missing indicator or separate class is straightforward and has demonstrated improved predictive performance over models omitting them.^17^ However, their inclusion could double the number of candidate predictors for a model. The approach also fails to capture complex representations of the measurement process.

#### Summary measures

An extension to missing indicators, capable of incorporating both IP and IO, is to derive a summary of the measurement process and include this as a predictor.^15^^,^^24^^,^^31–44^ Examples include a count of the number of measurements (eg, throughout a critical care admission),^37^ weighted counts,^42^ combined missing indicators,^31^ missingness rates over time,^32^ time intervals between measures,^33–35^ embedding vectors that represent missing values,^36^ or information relating to hospital processes.^38^^,^^39^

In some cases, combined missing indicators and time intervals also alter the relationship between a predictor and outcome. Che et al’s^24^ method stipulates that the longer a measure has been missing, the less influence it should have on an individual’s prediction; therefore, the last observed measurement is decayed toward a mean value.

Piecewise-constant intensity models have also been proposed to handle informatively observed predictors.^40^^,^^41^ Piecewise-constant intensity models use decision trees to assign an intensity rate to the observation process, conditional on its history (timings, values, and events).

Define a summary measure of the observation process Q, eg, a count of the number of times X(t) (whether continuous or categorical) has been observed:$\;Q=m_{i}$. For cross-sectional prediction with a time-varying covariate, we then have:

(3) $$g\left(P[Y=1|X,\;Z\right)=\beta_{0}+\;\beta_{1}X+\;\beta_{2}Z+\gamma Q$$

where X is a summary of X(t) deemed to have predictive value (eg, the mean, most recent, or most extreme value). If X(t) has never been observed, this should be imputed. Like missing indicators, summary measures are easily derived and implemented in any prediction model using standard software (since they are included as standard predictors). Combining missing indicators into one summary, or implementing a dimension-reduction technique such as Lasso, also overcomes the issue of including multiple missing indicators. However, selecting the most appropriate summary measure for a model requires careful consideration, and will depend on the clinical application. No current guidance exists on how best to choose the most appropriate summary measure. The association between a chosen summary measure and the outcome might lack generalizability where measurement processes vary across locations.^23^^,^^39^ Simple summary measures such as counts may also fail to capture the complex relationship between the observation process and outcome.

### Category 2: Modeling under IP

While the methods in the other categories can be used to handle both IP and IO, this category comprises methods that have specifically been proposed to handle IP.

#### Pattern-specific models

The pattern-specific approach^45^^,^^46^ derives separate models for each missingness pattern, generalizing the missing indicator approach. The model corresponding to the observed pattern in a new individual is then used for prediction. For example, in a model with a single partially-observed time-invariant continuous predictor, X we would derive the following submodels:

(4) $$g\left(P\left[Y=1|R=1,\;X,\;Z\right]\right)=\beta_{0,1}+\beta_{1,1}X+\beta_{2,1}Z$$

(5) $$g\left(P\left[Y=1\;|R=0,\;Z\right]\right)={\beta_{0,2}+\beta }_{2,2\;}Z$$

Where Z is completely observed. Note that formulas 4 and 5 can also be combined by including interaction terms with the missing indicator, illustrating how this approach extends the missing indicator method.

Similar submodels could be derived for categorical and continuous predictors. Saar-Tsechansky and Provost^45^ proposed using all available data to train each submodel, whereas Fletcher et al^46^ recommended that only individuals in each observed pattern be used in the derivation of that pattern’s submodel (also illustrated by Janssen et al).^60^ The latter approach does not require knowledge of the missingness mechanism.

The pattern-specific approach is flexible, as it can be applied to any form of prediction algorithm. However, a practical limitation is that the number of candidate submodels becomes intractable as the number of predictors increases.

#### Likelihood-based methods

A different approach assumes that missingness in the predictors is nonignorable, and incorporates this into parameter estimates via likelihood-based methods.^47^^,^^48^ The model formulation would take, for example, the same form as equation 2, with parameter estimates obtained according to estimation procedures detailed in the following examples. Escarela et al^47^ assumed a bivariate copula-based probability function for the missing covariates and the missingness mechanism. Kirkham^48^ instead applied the “method of weights,” which assumes a parametric model for the missingness mechanism and incorporates this into the maximum likelihood estimation of parameter estimates.

Escarela et al^47^ described how their missing not at random model can also be used to impute missing values. However, this does not remove the need to make untestable assumptions on the missing data mechanism.

### Category 3: Latent structures

#### Similarity measures

Patient similarity measures apply a sequencing algorithm to establish the alignment of 2 sequences of patient data (eg, longitudinal EHR data). Sha et al^49^ presented a novel similarity measure, which recognizes that the type of tests ordered and the time between tests can be indicative of patient condition. Their metric is therefore based on a distance measure incorporating the type, timings and results of tests and they assume that more intense monitoring indicates a more severe condition.

The sequencing algorithm produces a similarity matrix, defining the similarity between each pair of patients. We do not present the model formulation for this method since there are various approaches to using this matrix in prediction (described by Sharafoddini et al.)^61^ One such method defines cohorts of “similar” patients within which to develop separate models. This approach can be viewed as an extension of the pattern submodel approach with longitudinally and irregularly measured predictors, in which the patterns are defined by similar longitudinal sequences.

The benefit of this method is that, as with others, it can be applied to any form of prediction framework. Drawbacks include the computational burden of rederiving multiple models, and requiring access to the training data at prediction time to train a model using similar patients.

#### Latent variable

A simple way of representing a latent clinical condition is to use a single (partially) latent binary variable, representing 1 of 2 states. This approach was used by Coley et al^50^ and Hubbard et al^51^ in which IP and IO are incorporated by allowing the measurement process to infer a latent patient condition under a hierarchical structure.

Define the partially latent binary outcome $Y^{L}\;∼\;\mathrm{Bern}(\eta )$ representing 1 of 2 patient states, where only 1 state is entirely observed. In Coley et al^50^’s example, “true” cancer state (aggressive vs indolent) is the outcome, but is only observed for a subset of patients who underwent surgery. We then assume that the value of the outcome can influence the presence of $x_{i}$ within the hierarchical model.

(6) $$R\left|\;Y^{L},\;Z\;∼\;\mathrm{Bern}\left(P\left[R=1\;\right|\;Y^{L},\;Z,\;\beta \right]\right)$$

We have not provided the outcome model formulation since predictions are obtained by sampling from the posterior of the full hierarchical model.

Both studies note improved predictive performance in which the measurement process influences predictions compared with a model that ignores IP and IO. These models can, however, be computationally intensive to fit.

#### Hidden Markov models

Hidden Markov models extend the latent variable approach by allowing a time-varying latent process. Zheng et al^52^ and Alaa et al^53^ used hidden Markov models to capture IO, but the way they incorporated the observation process differs. Hidden Markov model–based prediction models incorporate IO by allowing the measurement frequency or rate to infer the clinical state at any given time.

Alaa et al^53^ proposed a latent semi-Markov process to capture a patient’s evolving clinical state. The “state” variable, $Y^{L}\left(t\right)\in \{1,\;\ldots ,\;4\}$, ranges from clinical stability to clinical deterioration, where stability (state 1) and deterioration (state 4) are observed states, but intermittent states are latent. Here the model aims to predict eventual clinical deterioration, that is, $P[Y\left(\infty \right)=4]$. The observation process (ie, timings) of X(t) is used to infer this clinical state, in which it is assumed that increased monitoring indicates a less stable condition. A marked point process model (in this case a Hawkes process) is adopted to model the rate of patient monitoring, with the marks corresponding to the observed value. IO is captured through state-specific intensity functions for the monitoring frequency as follows:

(7) $$\lambda (t\;|Y^{L}(t)=1)=\;\lambda_{1}+\;\alpha_{1}\sum_{\tau <t_{m}<t}e^{-\beta_{1}\left(t-t_{m}\right)}$$

…

(8) $$\lambda (t\;|Y^{L}(t)=4)=\;\lambda_{4}+\;\alpha_{4}\sum_{\tau <t_{m}<t}e^{-\beta_{4}\left(t-t_{m}\right)}$$

$\lambda_{1},\ldots ,\;\lambda_{4},\;\alpha_{1},\ldots ,\;\alpha_{4},\beta_{1},\ldots ,\;\beta_{4}$ are state-specific parameters to be estimated. $t_{m}$ is the time of the last measure of X(t). τ is the time of the most recent change in $Y^{L}(t)$, which is only observed if the state is absorbing. Details of the learning and prediction algorithm are presented in more detail in their article.

A key advantage is that the Hawkes process allows for a time-varying intensity in the observation process. Model fitting and interpretation are, however, complex because there are multiple components to be estimated simultaneously.

#### Joint modeling

Joint modeling has been developed extensively within the prediction context, particularly for dynamic prediction, that is, incorporating time-updated variables (S2 in Table 1).^62–65^ Joint modeling can be extended to handling IP and IO, by linking the outcome to the observation process via a shared random effect,^54–56^ which can be seen as an alternative approach to modeling latent variables. Separate models are defined for the outcome occurrence and the observation process, each of them containing an individual-level random effect representing individual “frailty.” By sharing these random effects across the 2 models, the outcome and observation processes are linked. Liang et al^54^ and Choi et al^56^ both allow for irregularly observed visits, and therefore specify a hazard or intensity function that defines how often visits occur. The random effect, or frailty term, controls how an individual’s visit rate differs from average. As this effect also appears in the model for the outcome, the visit rate indirectly affects the prediction for the outcome.

The method outlined in Zhang et al^55^ only allows for scheduled, regular observations. Therefore, rather than specifying a model for the intensity/hazard of visiting, the “observation process” model is a repeated measures logistic regression model, in which the outcome indicates whether an individual provided data at a specific time point.

Joint models take many different forms and provide the most general framework. We present an example of a trivariate joint model, with submodels for the repeatedly and informatively measured covariate, the binary outcome, and the observation process of the covariate$\;x_{ij}$. Assuming that measurement times are regular (, $t_{ij}=t_{j}\;\forall \;i,\;j$).

(9) $$X=\;\alpha_{0}+\;\alpha_{1}Z+\;\alpha_{2}t+\;U$$

(10) $$g\left(P[Y=1|Z,\;U,\;V]\right)=\beta_{1}Z+\beta_{2}U+\;\beta_{3}V$$

(11) $$h\left(P\left[R_{j}=1|U,\;V,\;Z\right]\right)=\delta_{0}U+V+\delta_{1}R_{j-1}+\;\delta_{2}Z$$

Here, U and V are independent subject-specific random effects, and $g\left(.\right)$ and h(.) are link functions. $\beta_{2}$ and $\delta_{1}$ control the relationships between the longitudinal predictor and the outcome, and the longitudinal predictor and the observation process, respectively. $\beta_{3}$ controls the association between the outcome and the missingness process. Missingness at time t depends on missingness at the previous measurement time.

The listed examples illustrate the flexibility of joint modeling, as the models for both the observation outcome processes can take different functional forms. Complex dependencies between the processes can be specified. However, fitting these models can be computationally intensive, and the interpretation of random effects in a prediction model can be challenging, especially for end users.^54^

## DISCUSSION

This study has identified 3 broad categories of approaches to incorporate IP or IO into clinical prediction models: derived predictors, modeling under informed presence, and latent structures. This is a growing area of research, and much of the included literature illustrates that IP and IO can be incorporated into clinical prediction models in a meaningful way. Where missing data and nonrandom visit processes have been seen as a nuisance in effect estimation, a more positive outlook is possible when the goal is prediction. Although methodology allowing CPMs to accommodate IP and IO are emerging, further challenges remain, which will be discussed later.

Pullenayegum and Lim^7^ and Neuhaus et al^9^ have previously reviewed methods for handling IO in studies in which the primary aim is to recover unbiased effect estimates. Both articles assume that the outcome is informatively observed, which differs from the focus of our work in which we assume informatively measured predictors. Phelan et al^5^ presented a set of design considerations for EHR-based studies that could help to attenuate issues caused by IP and IO by carefully considering and defining the population of interest (eg, in which part of the care system patient interactions occur) and how health status could affect patient interactions. None of these articles explicitly discuss prediction, in which we anticipate that the most appropriate methods will differ from those for effect estimation.

Empirical studies^37^^,^^66^ have compared methods capable of handling repeatedly measured predictors in CPMs, and many of these methods can be extended to accommodate IO, such as summarizing the process into a single measure (eg, the mean or maximum measurement patterns as predictors) or into more complex latent process methods. Both studies found that joint modeling provided little benefit in predictive performance when compared with simple summary measures, but care should be taken in selecting an appropriate summary measure suited to the clinical context. Bull et al^22^ also recommended 3 key considerations when choosing the most suitable method for harnessing a longitudinally measured predictor: the type and amount of information available at prediction time, how the CPM can benefit from the longitudinal information and the validity of assumptions for the particular application. We expect that these considerations will also be relevant to selecting the most appropriate means of incorporating IO.

To our knowledge, this is the first attempt at synthesizing the methodology available to handle IP and IO specifically for prediction purposes. We have achieved this through a systematic search of the literature. A potential limitation is that only the health and biomedical literature was considered; as such, our search potentially did not capture methods that have been developed for use in other fields. Defining relevant terminology around IP and IO is challenging, as the nomenclature differs across the literature. This is illustrated by the fact that a minority (n = 11 of 36) of included articles were discovered directly through database searches. However, this is a common challenge with methodological reviews.^67^^,^^68^ It is possible that methods were missed as a result, but we aimed to mitigate against this by conducting a backward and forward citation search on articles identified through the search strategy and on a set identified as relevant a priori.

Many of the methods discussed herein remain underdeveloped and future studies should investigate the degree to which these methodological choices matter for prediction contexts. We have identified multiple avenues for further research. Missing indicators, capable of handling both IP and IO, is the most common approach (in terms of number of studies included) to incorporating the observation process. Although this method is straightforward and adaptable to any type of prediction model, key challenges remain, including but not limited to the requirement to impute missing values when developing and applying the model. Under most prediction frameworks, a value must be entered for any predictor in the model when a prediction is made. The impact of using different imputation techniques at model development and prediction time should be established.

Pattern-specific models present a promising extension to the missing indicator approach, and do not require imputation at either model development or application. Further development should explore ways to borrow strength across models, or pool together sets of patterns, to overcome the issue of developing models with few data points for rarely observed missingness patterns.

Most methods capable of handling IO fall under the “summary measures” category (16 articles). The simplicity of this approach is attractive but is also a concern. Simple summaries of the entire process do not capture important changes in the observation process over time, such as a sudden increase in monitoring frequency which indicates worsening state. Latent structure approaches (eg, modeling measurement times via a nonhomogeneous point process) may be better suited to capturing longitudinal variability but are computationally intensive. Developing a more sophisticated representation of the observation process to use as a predictor is a promising avenue of further research, offering a potential trade-off between the simplicity of summary measures and the sophistication of joint modeling. These more complex measures should be compared with both joint modeling techniques and simple summary measures to assess their added benefit in terms of predictive performance and computational efficiency. We plan to perform such comparisons in a separate full empirical study.

There already exists a vast body of literature on joint modeling for prediction, particularly covering S2 (incorporating longitudinal predictors). Such methods have also recently been extended to functional data,^69^ allowing them to accommodate complex structures in longitudinal predictors. Joint models have also been proposed to handle IO under an inferential framework,^8^^,^^9^^,^^70^^,^^71^ so it follows that there is scope to extend joint models further to exploit IO for predictive benefit, as this review revealed that the method remains underdeveloped for this particular purpose.

There are broader challenges associated with exploiting IP and IO for prediction. First, because the association between the observation process and outcome is unlikely to be causal, this relationship may not generalize well to different settings. For example, clinicians’ monitoring behaviors are likely to vary across units or clinical guidelines could recommend changes in the way patients are observed. This is particularly true following the introduction of a CPM into clinical practice; once this happens, the predictor variables in the model are far more likely to be observed. The predictive utility of any model incorporating the observation process should therefore be regularly validated and potentially updated.

A second challenge described by Alaa et al^53^ concerns models that use the observation process to inform predictions, but also update predictions as new information becomes available. An issue arises when clinicians change their monitoring behavior based on predictions produced by the model; any changes in the way they monitor patients will be fed back into future predictions via the observation process. This should be accounted for to avoid the feedback loop, potentially by developing causal models to account for the possible time-varying confounding,^72^ or by explicitly modeling the effects of previous predicted values.

Despite these challenges, we view IP and IO as opportunities to improve the performance of predictive models, as opposed to a nuisance. The literature is divided on this point; much of the work in this review proposes methods that “overcome” the challenges of IP and IO, whereas others illustrate the added benefit of incorporating informative measurement patterns. Missing data have typically been seen as a threat to the estimation of parameters, but because this is not the key focus of prediction research, it may be useful to move away from terms such as *missingness* and instead focus on what the presence of an observation can tell us.

## CONCLUSION

We have demonstrated that there is a growing recognition of both IP and IO within prediction research. Although parallels exist with missing data, IP should not be considered the same way, especially within the context of prediction and routinely collected data in which there is no prespecified observation process. By synthesizing the available methods and software that could be applied to incorporate IO and IP into CPMs, this article can assist applied researchers in adopting suitable methods. Future research should investigate the challenges presented herein, which will require the development of formal guidelines and making existing methodology more accessible.

## FUNDING

This work was supported by Medical Research Council grants MC_UU_00002/5 (JKB), MC_UU_00002/2 (BT), and MR/N013751/11 (RS) and the Alan Turing Institute under the “Predictive Healthcare” project (Health and Medical Sciences Programme).

## AUTHOR CONTRIBUTIONS

RS designed the study, conducted screening, and wrote the manuscript. LL conducted screening and provided critical revisions to the final manuscript. GPM, MS, and NP provided substantial contributions to the conception, design and conduct of the work, and provided critical revisions to the final manuscript. JKB, BT, and KD-O contributed to discussions on study design and conduct, and provided critical revisions to the final manuscript.

## SUPPLEMENTARY MATERIAL

Supplementary material is available at *Journal of the American Medical Informatics Association* online.

## Supplementary Material

ocaa242_Supplementary_Data
