# Supplementary material for: Informative presence and observation in routine health data: A review of methodology for clinical risk prediction
Source: J Am Med Inform Assoc. 2020 Nov 9;28(1):155–66. doi: 10.1093/jamia/ocaa242 (PMC7810439; doi:10.1093/jamia/ocaa242)
Supplement: ocaa242_Supplementary_Data [file ocaa242_supplementary_data.docx]

# Supplementary material

## Appendix 1: Database Searches

Searches were tailored to each database to maximise specificity of the strategy. For each database, a strategy was defined to search for terms relating to informative presence and observation. These were then combined with the Geersing filter to restrict the search to prediction-related work.

## Web of Science IO & IP Strategy

| **Web of Science Search Terms – related to Informative Presence/Observation** |
| --- |
| TS = ((Informative* OR Informed OR Nonrandom OR Nonignorabl* OR Non-random OR Non-ignorabl*) NEAR/5 (Observation* OR Presence OR Absence OR Missing* OR Follow-up OR "follow up" OR completeness OR sampl* OR nonresponse OR non-response OR drop-out OR dropout)) |
| TS = ("Observation process") |
| TS = (“Visit* process”) |
| TS = (“Visit* pattern”) |
| TS = ("Inconsistently collected data") |
| TS = (MNAR) |
| TI = ("Missing not at random") |

## Ovid (MEDLINE & Embase) Search Terms

| **Terms related to observation processes** |
| --- |
| Inform* presence |
| Inform* observ* |
| Observ* process |
| Inform* missing* |
| Inform* follow up |
| Inform* sampl* |
| Irregular* sampl* |
| Non random sampl* |
| Non random completeness |
| Inform* completeness |
| Inconsistently collected data |
| Visit* process |
| Visit* pattern |
| MNAR |
| Missing not at random^†^ |
| Non ignorable missingness |

## Geersing Search Filter

The set of terms related to IP/IO was combined with the Geersing filter,[21] which has shown good sensitivity in detecting literature related to prediction model research. This filter was adapted to the required syntax for each database, and combined with the IO/IP terms specified in the previous two sections. The filter has showed good sensitivity in picking up CPM research.

Some flexibility was allowed in the definition of the search strategy, due to uncertainty in the types of studies that would be returned. Terms were revisited and collapsed/expanded during the early phases of the first screening stage.

| **Ingui CPM Search Strategy + Geersing Update** |
| --- |
| (Validat* OR Predict*.ti. OR Rule*) OR (Predict* AND (Outcome* OR Risk* OR Model*)) OR ((History OR Variable* OR Criteria OR Scor* OR Characteristic* OR Finding* OR Factor*) AND (Predict* OR Model* OR Decision* OR Identif* OR Prognos*)) OR (Decision* AND (Model* OR Clinical* OR Logistic Models)) OR (Prognostic AND (History OR Variable* OR Criteria OR Scor* OR Characteristic* OR Finding* OR Factor* OR Model*)) OR Stratification OR ROC Curve OR Discrimination OR Discriminate OR c-statistic OR c statistic OR Area under the curve OR AUC OR Calibration OR Indices OR Algorithm OR Multivariable |

## Appendix 2: Justification of Exclusion criteria

We acknowledge that selection bias and censoring are concepts related to IP and IO. However we were primarily interested in the context where the sample accurately represents the population of interest, but the informativeness of the observation process of predictors is of primary interest. Non-medical literature was not considered, and while methods to incorporate IP/IO may exist within other fields, our primary interest is their handling within health research, and more specifically in EHRs. Imputation methods have been omitted since, under informative observation, imputing data is non-trivial, as there are no predefined time points at which data should be imputed[7] with the typical irregular patient/clinician-driven visit processes that characterises informative observation. Moreover, imputing data risks losing important information, by making all patients appear to have been monitored at equal intervals.

## Appendix 3: Snowballing Set

A forward and backward citation search was performed on the following list of papers. These have all been deemed relevant to informative observation, but do not all meet the inclusion criteria for this review.

- Weiskopf, Nicole G, Alex Rusanov, and Chunhua Weng. 2013. “Sick Patients Have More Data: The Non-Random Completeness of Electronic Health Records.” *AMIA ... Annual Symposium proceedings. AMIA Symposium* 2013: 1472–77. http://www.ncbi.nlm.nih.gov/pubmed/24551421 (September 27, 2018).
- Rusanov A, Weiskopf NG, Wang S, Weng C. Hidden in plain sight: bias towards sick patients when sampling patients with sufficient electronic health record data for research. *BMC Med Inform Decis Mak*. 2014;14(1):51. doi:10.1186/1472-6947-14-51
- Phelan, Matthew, Nrupen A Bhavsar, and Benjamin A Goldstein. “Illustrating Informed Presence Bias in Electronic Health Records Data: How Patient Interactions with a Health System Can Impact Inference.”
- Sperrin, Matthew, Emily Petherick, and Ellena Badrick. 2017. “Informative Observation in Health Data: Association of Past Level and Trend with Time to Next Measurement.” *Studies in health technology and informatics* 235: 261–65. http://www.ncbi.nlm.nih.gov/pubmed/28423794 (June 25, 2018).
- Agniel D, Kohane IS, Weber GM. Biases in electronic health record data due to processes within the healthcare system: retrospective observational study. *BMJ*. 2018;361:k1479. doi:10.1136/BMJ.K1479
- Pivovarov R, Albers DJ, Sepulveda JL, Elhadad N. Identifying and mitigating biases in EHR laboratory tests. *J Biomed Inform*. 2014;51:24-34. doi:10.1016/J.JBI.2014.03.016
- Haneuse S, Daniels M. A General Framework for Considering Selection Bias in EHR-Based Studies: What Data Are Observed and Why? *EGEMS (Washington, DC)*. 2016;4(1):1203. doi:10.13063/2327-9214.1203
- Goldstein BA, Navar AM, Pencina MJ, Ioannidis JPA. Opportunities and challenges in developing risk prediction models with electronic health records data: a systematic review. *J Am Med Informatics Assoc*. 2017;24(1):198-208. doi:10.1093/jamia/ocw042
- Fletcher Mercaldo S, Blume JD. Missing data and prediction: the pattern submodel. *Biostatistics*. September 2018. doi:10.1093/biostatistics/kxy040
- Lin, Jau-Huei, and Peter J. Haug. 2008. “Exploiting Missing Clinical Data in Bayesian Network Modeling for Predicting Medical Problems.” *Journal of Biomedical Informatics* 41(1): 1–14. https://www.sciencedirect.com/science/article/pii/S1532046407000524?via%3Dihub (October 8, 2018).

## Appendix 4: paper-level summary of included articles

| **Author(s)** | **Year** | **Title** | **Broad group** | **Category** | **IP or IO** | **Description of method for incorporating IO/IP** |
| --- | --- | --- | --- | --- | --- | --- |
| Liang et al. | 2018 | Bayesian nonparametric inference for panel count data with an informative observation process. | Latent structures | Joint modelling | Both | - Cumulative count outcome e.g. recurrent events (tumour recurrence) - predicting future disease recurrences.  - Bivariate joint model for panel count data when observation process and event processes are dependent. - Nonhomogeneous Poisson processes for event process and observation process - Stationary Gaussian processes for baseline functions of the two processes - Processes linked via correlated frailty terms, following a bivariate lognormal distribution. |
| Che et al. | 2018 | Recurrent Neural Networks for Multivariate Time Series with Missing Values. | Derived predictors | Missing indicator, summary measures | IO | -Takes multivariate time series (longitudinal predictors) data to predict diagnoses and mortality, as binary outcomes. - Uses a form of Recurrent Neural Network called the Gated Recurrent Unit. - Uses both presence/absence of predictors ("masking") and time intervals between measures as inputs in RNN. - Also allows the influence of predictors to decay over time when they have been missing for a while. - Allows for different decay rates for each predictor, to be learned from the data. |
| Coley et al. | 2017 | A Bayesian hierarchical model for prediction of latent health states from multiple data sources with application to active surveillance of prostate cancer. | Latent structures | Latent variable | IP | - Bayesian hierarchical model that predicts an individual's underlying health state via joint modelling of repeated PSA measures and biopsies. - Predictions are informed by a subset of patients for whom the true state is actually observed (those who underwent prostatectomy). Therefore prediction target "cancer state" is partially latent. - PSA (continuous predictor) is modelled using a multilevel model, with random effects (intercept and age effect) varying across latent states. - Biopsy occurrence modelled as logistic regression (binary indicator of biopsy vs no biopsy) within regular time intervals. |
| Sengupta et al. | 2017 | Prediction and imputation in irregularly sampled clinical time series data using hierarchical linear dynamical models. | Derived predictors | Summary measures | IO | - Authors develop Kalman filters that explicitly model the time difference between two measures, capturing the dependency between clinical variables and the measurement times. - The state at a given time is allowed to depend on the previous state and the time instant at which the previous observation was made.  - Outcomes are all continuous physiological variables. |
| Zhang et al. | 2013 | A joint model of binary and longitudinal data with non-ignorable missingness, with application to marital stress and late-life major depression in women | Latent structures | Joint modelling | Both | - Predicting binary primary endpoint: probability of having Major Depressive Disorder (MDD), given individual trajectory of marital stress and an informative missing data mechanism. - Three components of the Shared Parameter Model: 1) Linear Mixed Model for longitudinal measures of marital stress, 2) GLM for binary primary endpoint (MDD), and 3) Shared parameter logistic regression model for the missingness mechanism - Subject-specific random effect shared across all models. - Include missingness at the previous visit as a predictor in missingness at current visit to account for dependence on prior missingness. |
| Escarela et al. | 2016 | Addressing missing covariates for the regression analysis of competing risks: Prognostic modelling for triaging patients diagnosed with prostate cancer | Modelling under informed presence | Likelihood-based methods | IP | - Likelihood-based method for estimating parameters under MAR and MNAR missingness in two categorical covariates. - Competing risks outcome, so mixture model is used. - They use a copula formulation for the covariate model and missing data mechanism. |
| Helander et al. | 2015 | Time-series modeling of long-term weight self-monitoring data. | Derived predictors | Missing indicator/summary measures | IO | - The goal is to predict future Weight, given a set of past weight data (time-series data). - The authors note that absence of weight data on a previous day predicts absence of data on the next day. - They build an ARIMA model to predict future weight, and incorporate absence flags for the M previous days in the model. M was varied between 0 and 15 days, and the best value chosen on the basis of AIC. For one subject a value of M = 3 was selected, for the other, a value of M = 9. |
| Barclay et al. | 2014 | Chain Event Graphs for Informed Missingness | Derived predictors | Separate class | IP | - A form of tree-based method, which incorporates missingness as a separate "event" in the Chain Event Graph, allowing for it to be informative of outcome.  - By exploring predictions made under MAR and MNAR assumptions, the method allows us to assess plausibility of the MAR assumption. |
| Kirkham | 2008 | A comparison of hospital performance with non-ignorable missing covariates: An application to trauma care data | Modelling under informed presence | Likelihood-based methods | IP | - Outcome is 30-day survival following trauma as a dichotomous variable. - The method used to handle missing covariates is the "method of weights" in generalized linear models. - They adapt the work of Joseph Ibrahim, who proposed a ML based approach using the EM algorithm assuming a nonignorable missing mechanism. - The author anticipates that under many settings, missingness is related to the condition of the patient and therefore nonignorable (NMAR), and failure to observe depends on the values that would have been observed. |
| Alaa et al. | 2017 | Learning from clinical judgments: semi-Markov-modulated marked Hawkes processes for risk prognosis | Latent structures | Hidden (semi-) Markov Models | IO | - Method designed to account for an incorporate the fact that the frequency of patient monitoring in inpatient care is dependent on the patient's latent clinical state. - They propose representing the monitoring scheme as a marked Hawkes process. Intensity of the point process is defined by intensity parameters which depend on patient state (latent), and the observed physiological data are modelled using a switching multi-task Gaussian process. - Patient latent clinical states are represented as an absorbing semi-Markov jump process (absorbing to reflect the fact that episodes are informatively censored). - The target of prediction, and a patient's risk score at any time, is taken to be the probability of eventual absorption into a "clinical deterioration" state. |
| Zheng et al. | 2017 | Resolving the bias in electronic medical records | Latent structures | Hidden (semi-) Markov Models | IO | - This method recognises that EMR data are essentially an irregular time series, with irregular visiting times and different diagnoses/tests recorded at each visit. The goal is to transform it into a regular time series which is easier to analyse.  - A multivariate time-series with regular intervals is created, and the hidden condition at each regular time point is learned, but uses the informative observation process to infer the hidden states. The goal is to then use methods developed for regular time series on the transformed series. - Authors define their specific type of bias as the fact that 1) patients visit hospital more often when sick and 2) doctors order lab tests that are likely to be abnormal. - A "hidden condition" is defined at each time point, which is inferred by how and whether patients with particular conditions are observed frequently. - They define the "observation rate" as "the probability of one medical feature being observed at a time point, based on its actual condition (e.g. present/absent, negative/normal)." |
| Islam et al. | 2017 | Marked Point Processes for Severity of Illness Assessment | Derived predictors | Summary measures | IO | - Prediction of mortality in the ICU from noisy, incomplete, heterogeneous, unevenly sampled patient data. - This paper fits a Piecewise Constant Conditional Intensity Model - a non-Markovian marked point process to model irregular observation streams in continuous time.  - The PCIM point process can be expressed as decision tree, with internal nodes (e.g. "time between t-1 and t-5?") the binary test functions, and leaves as the "states", which define the intensity rate (easiest to visualise this - see diagrams in paper). - They learn separate PCIM models for patients who died, and those who did not. The log odds of the two models is then used as a severity score feature for individuals, and entered into a SVM classifier as a feature. |
| Lipton et al. | 2016 | Modeling Missing Data in Clinical Time Series with RNNs | Derived predictors | Missing indicator/summary measures | IO | - Goal of prediction model is multilabel classification; choosing from a set of 128 possible diagnoses, where each patient can experience more than one. - They aim to model missingness directly as a binary indicator, as the authors note that which tests were ordered can be more predictive than the results of said tests. - They also compare missingness indicators in combination with Zero Imputation and Forward Filling (LOCF) imputation techniques. The justification for LOCF is that items are likely to be measured when clinicians believe there has been a change in the value, and remain the same otherwise. - They compute a range of features related to missingness of individual items for use in the logistic regression model only, since a linear model "can only learn hard substitution rules".  - They find that all models improve when either indicators or the manually computed features are included in the model, but this improvement is more modest in the logistic regression case. |
| Ghorbani & Zou | 2018 | Embedding for Informative Missingness: Deep Learning With Incomplete Data | Derived predictors | Summary measures | IP | - The aim is to provide a general framework for training neural network predictors when the training data has missing features. - The authors propose a flexible embedding method that learns a representation for missingness directly from the data. - The method does not require any imputation, and can handle informative missingness. |
| Li & Xu | 2019 | VS-GRU: A Variable Sensitive Gated Recurrent Neural Network for Multivariate Time Series with Massive Missing Values | Derived predictors | Missing indicator/summary measures | IO | - The proposed method is called variable sensitive GRU (VS-GRU). It considers the missingness rates of each variable individually rather than as a whole. - For each variable at each time point, create a missingness indicator to differentiate between imputed and observed values. Also calculate the missing rate of each predictor. - The missing indicators for each individual variable as used as inputs/features in the VS-GRU model, as well as the missing factor (defined in the next column). |
| Rodenburg et al. | 2019 | Improving RNN Performance by Modelling Informative Missingness with Combined Indicators | Derived predictors | Missing indicator/summary measures | Both | - The method proposes summing missing indicators to avoid the issue of potentially doubling the number of predictors in a model where using a single missing indicator for each predictor. |
| Sharafoddini et al. | 2019 | A New Insight Into Missing Data in Intensive Care Unit Patient Profiles: Observational Study | Derived predictors | Missing indicator/summary measures | IP | - Uses simple missing indicators to predict patient mortality in the ICU. - Each patient's data was summarised over every day of their admission, with indicators representing which lab tests were ordered in a particular day. - The missing indicators were added to the predictor matrix to create an augmented dataset. Missing values were imputed using Hot Deck and predictive mean matching single imputation techniques. - Feature selection methods were employed to select the most informative missing data indicators. - They attempt fitting a model on missing indicators alone, and find fairly good predictive performance. However they note that these models would not be sufficient for use in clinical practice. |
| Lin & Haug | 2008 | Exploiting missing clinical data in Bayesian network modeling for predicting medical problems | Derived predictors | Missing indicator/separate class | IP | - The method explicitly represents missing items in a clinical decision system to improve predictive performance.  - All methods are a form of Bayesian Network used to predict diagnoses; a naïve Bayes structure, a human-composed network structure, and two networks based on structural learning algorithms. - They compare different ways of incorporating (or ignoring) information in the missingness, and find that those methods explicitly modelling missing items perform best.  - Missingness is represented as either a separate class or a separate indicator variable. |
| Badgeley et al. | 2019 | Deep learning predicts hip fracture using confounding patient and healthcare variables | Derived predictors | Summary measures | IP | - Hospital process variables (related to image acquisition) are added as predictors in a model. - Variables considered are: department, scanner model, scanner manufacturer, laterality, study date (and day of week), order priority, technician, radiologist, radiation dose, time from image order to acquisition, time from image acquisition to initial interpretation, time from image acquisition to final interpretation. - They fit: logistic regression models and convolutional neural networks - Most hospital process vars were found to be statistically significantly associated with fracture (p < 0.05) - Missing items were imputed |
| Zhang et al. | 2019 | Healthcare processes of laboratory tests for the prediction of mortality in the intensive care unit: a retrospective study based on electronic healthcare records in the USA | Derived predictors | Summary measures | IO | - Similar to the Badgeley et al paper; this time predicting mortality in the ICU using variables related to the collection of lab tests. - Process variables this time are: the clock hour, the number of measurements and the measurement time from ICU admission - GLMs (logistic regression) are fitted with hospital mortality as the outcome. - AUROC increased with addition of the process variables. |
| Sha et al. | 2016 | A Novel Temporal Similarity Measure for Patients Based on Irregularly Measured Data in Electronic Health Records | Latent structures | Similarity measures | IO | - Authors create a patient similarity measure which incorporates the ordering and time intervals between lab tests. - They hypothesise that the timestamps, order, and frequency of measurements in addition to the results could carry meaningful information about patient condition. - Their similarity is novel since it incorporates time-varying information, as well as information on e.g. time intervals between tests. - The measure takes only the 10 most commonly ordered lab tests from each dataset used (MIMIC-II and CHOA). - Their novel similarity measure is compared against two non-temporal similarity measures, and find improved predictive performance. - The similarity measure is used to define patient cohorts within which to develop separate models. |
| Hubbard et al. | 2018 | A Bayesian latent class approach for EHR‐based phenotyping | Latent structures | Latent variable | IP | - Develops a method that can handle informatively missing predictors using a Bayesian latent class approach in a phenotyping context. - This is an unsupervised learning method, where the true gold standard phenotype is not available for any patients. - Method assumes that true disease state is unavailable, but may influence which data are available for an individual. - The approach appears to perform well, even under MNAR. - Prior knowledge about classification accuracy of biomarkers and codes can be incorporated through suitable choice of priors. |
| Goldstein et al. | 2017 | A comparison of risk prediction methods using repeated observations: an application to electronic health records for hemodialysis | Derived predictors | Summary measures | IO | - This paper primarily compares methods that generally allow for repeated biomarker measurements in a prediction model.  - However, they explore the incorporation of informed presence, by adding in the number of times a measurement is taken as a predictor. - Authors comment on the predictability of the number of measurements taken as a simple summary statistic. However they note that this is only the case for the vital signs, as these are not measured on a scheduled basis as labs would be in this setting. |
| Fletcher Mercaldo & Blume | 2018 | Missing data and prediction: the pattern submodel | Modelling under informed presence | Pattern-specific models | IP | - Develops separate models for each missingness pattern: the pattern submodel (PS) - Therefore accommodates missing data at both model development and prediction time, and does not require any imputation at development or prediction time. - The key difference with regular pattern mixture models is that only data in the observed patterns are used to develop the models; this means a that no assumptions must be placed on the missing data mechanism - The paper focuses on assessing performance at prediction time, comparing the pattern submodel with commonly applied imputation techniques. |
| Fauber & Shelton | 2018 | Modeling “Presentness” of Electronic Health Record Data to Improve Patient State Estimation | Derived predictors | Point processes | IO | - Uses Piecewise-Constant Intensity Models to build a generative model of observation times and values.  - The model is used to predict future values of vital signs based on the history of these events. - They note that data are rarely MAR in medical settings, and instead that the frequency or absence of events should be used to estimate patient state. - An existing PCIM model is extended to incorporate not only the rate of events, but also values. |
| Zabihi et al. | 2019 | Sepsis Prediction in Intensive Care Unit Using Ensemble of XGboost Models | Derived predictors | Summary measures | IO | - Authors note that the pattern of missing data may convey useful information, and should therefore be used to aid prediction.  - Missing data are first imputed and summary measures are computed to be used as features in the model. - Authors define sequence abstraction: each sequence is defined as a set of consecutive measures where values are either missing or present, e.g. SBP measures for a 6 hour period (1 hour intervals): {NA, 122, 98, NA, NA, 123}. Based on their definition, we have four sequences of {NA}, {122,98}, {NA, NA} and {123}. Sequence abstraction calculates and uses as features: 1) Mean and variance of the lengths of sequences along each covariate. 2) Summation and variance of the lengths of sequences with only valid values (no missing) along each covariate, and 3) Mean and variance of the lengths of sequences along each observation, in the last 5 hours. - These features representing different aspects of the missingness patterns are entered into a classifier. |
| Du et al. | 2016 | Recurrent Marked Temporal Point Processes: Embedding Event History to Vector | Derived predictors | Marked point processes | IO | - Proposes 'Recurrent Marked Temporal Point Processes' (RMTPP) to simultaneously model event timings and markers. - They aim to predict the time and type of future events from the history of a sequence of many events. - The key idea of the approach is to view the intensity function of a temporal point process as a nonlinear function of the history of the process, and parameterize the function using a recurrent neural network. - Using our model, event history is embedded into a compact vector representation which can be used for predicting the next event time and marker type. - Based on the hidden unit of RNN, we are able to learn a unified representation of the dependency over the history. |
| Choi et al | 2019 | Joint nested frailty models for clustered recurrent and terminal events: An application to colonoscopy screening visits and colorectal cancer risks in Lynch Syndrome families | Latent structures | Joint modelling | IO | - Authors introduce and use a joint nested frailty model (JNFM) to predict risk of colorectal cancer (CRC).  - They incorporate the visit process of screening visits as a recurrent events process and cancer occurrence (prediction target) as a terminal event. - Model provides dynamic predictions, allowing predictions to update as new information becomes available. - They allow for an individual-specific frailty which links the processes. - Their data also contains clustering at the family level, which is included in the model via another frailty term for each family.  - The number of screening visits per individual is highly irregular, and the timing between visits varies both within and between individuals. |
| Jarrett et al | 2019 | Dynamic Prediction in Clinical Survival Analysis using Temporal Convolutional Networks | Derived predictors | Missing indicators | Both | - Authors are proposing Match-Net: a Missingness-Aware Temporal Convolutional Hitting-time Network. Designed to capture temporal dependencies and heterogeneous interactions in covariate trajectories and patterns of missingness. - The model makes no assumptions regarding the underlying longitudinal or time-to-event processes. - The model can provide dynamically updated survival predictions, as well as accommodating informative patterns of missingness. - The model explicitly accounts for informative missingness by learning correlations between patterns of missingness and disease progression. - The model accounts for potential informativeness of both irregular sampling (intervals between consecutive visits and measures may vary) and asynchronous sampling (not all features are measured at the same time or same frequency). |
| Saar-Tsechansky | 2007 | Handling Missing Values when Applying Classification Models | Modelling under informed presence | Pattern-specific models | IP | - Key method of interest here is the "reduced models" approach, where separate models are developed for different missingness patterns (as described later by Fletcher Mercaldo). -Proposes developing a separate model for each missingness pattern, but using all available data, NOT just those observed within the pattern (as with Fletcher-Mercaldo's more recent paper). - Authors also propose a workaround for the possibility of having to develop huge numbers of models when p is large - develop models for "important" patterns, and using "lazy learning" or imputation for less important patterns. |
| Ding & Simonoff | 2010 | An Investigation of Missing Data Methods for Classification Trees Applied to Binary Response Data | Derived predictors | Separate class | IP | - Authors find that "separate class" (adding in an additional category for missing values) is the best method to use when the training set contains missing values and missingness is related to the outcome. - All methods here are considered under a classification tree framework, but also extended to logistic regression. Predictors must be categorised in tree-based methods, so the separate class works well here. - They also study different methods in a logistic regression model: missing indicator method, separate models for data with/without missing data (by-group method), imputing missing values with mean/mode and complete case. Missing indicator and separate models observations with/without missing values are the same as the separate class method in tree methods. |
| Bagattini et al | 2019 | A classification framework for exploiting sparse multi-variate temporal features with application to adverse drug event detection in medical records | Derived predictors | Summary measures | IO | - Provides a framework for using multivariate time series data to detect adverse drug events, considering that the sparsity in the available data may be useful in determining the existence of an ADE. - Proposes and compares three different methods for handling sparsity, one of which explicitly exploits it. |
| Wu et al | 2018 | Modeling Asynchronous Event Sequences with RNNs | Derived predictors | Missing indicators/summary measures/time intervals | IO | - Discusses different ways of measuring time, and of incorporating this into RNNs, .e.g time between events, time since a landmark event, burstiness of events. - Then establishes how this information should be used in RNNs; either concatenated into the predictor matrix, or used to mediate the importance of an input. i.e. the longer something has been unobserved, the less important it is. |
| Zhao et al | 2015 | Handling Temporality of Clinical Events for Drug Safety Surveillance | Derived predictors | Summary measures | IO | - This method handles informative observation (longitudinally measured predictors) by proposing different ways of counting the number of measures (or clinical events) that occur.  - The setting is in detecting Adverse Drug Events (ADEs), which are not necessarily recorded in the patient record. - The first method (Bag of events - BE) simply counts the number of times a measure occurs within D days. - Bag of Binned Events (BBE) counts the number of occurrences of each x in each day within D days. So each day has a separate feature calculated. - Bag of Weighted Events (BWE) assigns different weights to event x that occurred at different days d, and takes into account the weights when counting the number of occurrences of x. Weights are assigned according to the time distance between the event and the target ADE (prediction target). Those further away from the target ADE receive proportionally less weight. |
| Twala et al | 2008 | Good methods for coping with missing data in decision trees | Derived predictors | Separate class | IP | - Proposes "missingness incorporated in attributes" and compares against competing methods. - Method is very similar to separate class, but has also been extended for use in continuous predictors, where missingness can be used as the basis of a split in a tree-based model. |
|  |  | Biases in electronic health record data due to processes within the healthcare system: retrospective observational study | Derived predictors | Summary measures | Both | Explores the predictive ability of time of day, day of the week, and time between measures on mortality in inpatient admissions. Shows that the timing is a more accurate predictor of mortality than the result itself of some blood tests. |
| Agniel et al | 2018 |  |  |  |  |  |
